# Effect of Cannabis Legalization in Canada on the Incidence of Psychosis Consultations in Quebec City's Psychiatric Emergency Services

**DOI:** 10.1177/07067437241232901

**Published:** 2024-02-21

**Authors:** Sophie L’Heureux, Maxime Huot-Lavoie, Audrey Bergeron, Christina Bergeron, Bruno-Pier Blouin, Marc-André Roy

**Affiliations:** 1 PPEP Capitale-Nationale, Centre Intégré Universitaire de Soins et Services Sociaux (CIUSSS) de la Capitale Nationale, Québec, QC, Canada; 2 Department of Psychiatry and Neurosciences, 12369 Faculty of Medicine, Laval University, Québec, QC, Canada; 3 Centre Intégré de Santé et de Services Sociaux de Lanaudière, Joliette, Québec, Canada

**Keywords:** cannabis, cannabis legalization, psychosis, emergency, Quebec, first-episode psychosis

Canadian government legalized cannabis on October 17, 2018, and the Province of Quebec established the government-owned Société Québécoise Du Cannabis (SQDC) as the sole authorized distributor. Studies investigating the relationship between cannabis legalization and psychosis are limited and have yielded mostly inconclusive results.^1,2^ A nationwide study in the United States observed no significant increase in psychosis diagnoses in states where cannabis was legalized.^3^ A study in Alberta and Ontario observed such an increase but interrupted time series analyses suggested that this was not due to cannabis legalization as it was the continuation of a trend antedating legalization.^4^ In Quebec, an observational study at Sherbrooke revealed a significant increase in cannabis use following legalization but no significant difference in consultations for psychosis.^5^

The present retrospective observational study examined the impact of cannabis legalization on the incidence of consultations for cannabis-related psychotic episodes in the adult population, by comparing the proportion of emergency department (ED) consultations for psychoses in which evidence for cannabis consumption was obtained before and after legalization. Comparatively to the Sherbrooke study, it assessed a longer observation period and focused on the adult population, considering the legal age of 18 years old for purchasing cannabis until January 1, 2020. This study included patients seeking psychiatric consultation for psychosis at one of the three psychiatric EDs in Quebec City, within the 12 months preceding legalization of cannabis (October 17, 2017–October 16, 2018) and the 12 months following legalization (October 17, 2018–October 16, 2019). Medical records of adults with an active episode of psychosis notwithstanding the diagnosis (substance-induced psychosis, schizophrenia spectrum disorder, depressive and bipolar disorders) were reviewed by three psychiatry residents. Individuals >65 years old and cases of only substance intoxication were excluded. The occurrence of >1 consultation within the same month was considered as a single episode as recommended in the Diagnostic and Statistical Manual of Mental Disorders, Fifth Edition. Evidence of cannabis consumption came from an exhaustive review of medical records, including drug urine screening and clinical notes. Ethical approval was provided by the Research Ethics Committee of the CIUSSS-Capitale-Nationale. One-tailed chi-square test with a *P* ≤ 0.05 threshold for statistical significance was used to compare the proportion of ED consultations for psychoses with evidence of cannabis consumption before and after legalization.

## Results

A total of 2448 consultations for a psychotic episode (1254 before legalization and 1194 after) were analyzed (Table 1). We observed no differences regarding the gender and age distribution before versus after legalization nor any difference in the proportion of cases with cannabis consumption (36% before vs. 37.7% after). Comparisons of this proportion in subgroups defined according to gender or age yielded no significant differences although there were small increases within the 18–30 age subgroup (cannabis use = 55.0% before legalization vs. 61.3% after; *P* = 0.08). Among first-episode psychosis (FEP), there was also a small but nonsignificant increase in proportion (42.9% before, 47.9% after, *P* = 0.29). Interestingly, there was an increase in the percentage of FEP postlegalization (16.9% before, 21.5% after, *P* = 0.02) and in the percentage of FEP cases with cannabis use (7.3% before, 10.3% after, *P* = 0.05).

**Table 1.** Emergency Department Consultations for a Psychotic Episode Before and After Cannabis Legalization.

	*N* (% of total sample)	Before legalization	After legalization	Difference in proportion: after versus before; odds ratio	*P*-value
**Comparisons of % of cases with or without cannabis prior to versus after legalization in the full sample**					
Without cannabis use	1547 (63.2%)	803 (64.0%)	744 (62.3%)		
With cannabis use	901 (36.8%)	451 (36.0%)	450 (37.7%)	1.7%; 1.08	0.38
Total	2448	1254	1194		
**Comparisons of % of cases with or without cannabis prior to versus after legalization in the subsample of first-episode psychosis**					
Without cannabis use	255	121 (57.1%)	134 (52.1%)		
With cannabis use	214	91 (42.9%)	123 (47.9%)	5.0%	0.29
Total	469	212	257		

The present study observed no increase in the proportion of ED consultations for a psychotic episode in which evidence for cannabis consumption was obtained before and after legalization, which is in line with previous studies stating that legalization had no significative impact on ED's consultations for psychosis.^3–5^ The following issues should be considered while interpreting these results. First, the information on cannabis use relied exclusively on information gathered in the medical records, which may lead to some misclassification as nonusers; however, as this limitation was present prior to and after legalization, it is an unlikely explanation for the lack of change in proportion. Second, while the present sample was sizeable (total *n* = 2448), it might have lacked statistical power to detect a small impact of cannabis legalization, even more so in subsequent comparisons within patient subgroups, for example, FEP. Third, the one-year duration of the postlegalization period examined did not allow for examination of the longer-term impact of legalization, being potentially affected by (1) increased awareness of psychosis in the media only after legalization; (2) inconsistent supply and variable opening hours of SQDC in the first months; (3) availability of very different Δ^9^-tetrahydrocannabinol (THC) concentrations at SQDC. In conclusion, considering these methodological issues, the present results do not suggest that cannabis legalization had a major impact on the number of ED consultations for psychotic disorders. This could be explained by a limited augmentation of cannabis consumption in regular users in Quebec, by the delay between consumption and impact on psychosis or by the impact of the media awareness campaign and/or access to cannabis with lower THC concentrations. While this report does not suggest that ED visits for psychosis have been impacted significantly by the legalization of marijuana, further research is needed to determine whether cannabis legalization will have an impact on the incidence of first-episode psychosis.
